# SARS-CoV-2 Infection Causes Heightened Disease Severity and Mortality in a Mouse Model of Down Syndrome

**DOI:** 10.3390/biomedicines12030543

**Published:** 2024-02-28

**Authors:** Roger D. Pechous, Priyangi A. Malaviarachchi, Zhuo Xing, Avrium Douglas, Samantha D. Crane, Hayley M. Theriot, Zijing Zhang, Alireza Ghaffarieh, Lu Huang, Y. Eugene Yu, Xuming Zhang

**Affiliations:** 1 Department of Microbiology and Immunology, University of Arkansas for Medical Sciences, Little Rock, AR 72205, USA; 2 The Children’s Guild Foundation Down Syndrome Research Program, Department of Cancer Genetics and Genomics, Roswell Park Comprehensive Cancer Center, Buffalo, NY 14263, USA; 3 Rockefeller Cancer Institute, University of Arkansas for Medical Sciences, Little Rock, AR 72205, USA; 4 Departments of Ophthalmology and Pathology, University of Arkansas for Medical Sciences, Little Rock, AR 72205, USA

**Keywords:** SARS-CoV-2, COVID-19, Down syndrome, mouse model, pathogenesis

## Abstract

Recent epidemiological studies suggest that individuals with Down syndrome are more susceptible to SARS-CoV-2 infection and have higher rates of hospitalization and mortality than the general population. However, the main drivers behind these disparate health outcomes remain unknown. Herein, we performed experimental infections with SARS-CoV-2 in a well-established mouse model of Down syndrome. We observed similar SARS-CoV-2 replication kinetics and dissemination in the primary and secondary organs between mice with and without Down syndrome, suggesting that both groups have similar susceptibilities to SARS-CoV-2 infection. However, Down syndrome mice exhibited more severe disease as defined by clinical features including symptoms, weight loss, pulmonary function, and survival of mice. We found that increased disease severity in Down syndrome mice could not be attributed solely to increased infectivity or a more dramatic pro-inflammatory response to infection. Rather, results from RNA sequencing suggested that differences in the expression of genes from other physiological pathways, such as deficient oxidative phosphorylation, cardiopulmonary dysfunction, and deficient mucociliary clearance in the lungs may also contribute to heightened disease severity and mortality in Down syndrome mice following SARS-CoV-2 infection.

## 1. Introduction

Down syndrome is a genetic disorder characterized by trisomy for all or part of chromosome 21. People with Down syndrome are significantly predisposed to certain medical conditions including congenital heart defects, obstructive sleep apnea, diabetes, obesity, and Alzheimer’s disease, and Down syndrome is often associated with immune dysregulation including autoimmune diseases and immunosenescence [1,2,3]. At the molecular and cellular levels, people with Down syndrome have elevated levels of many inflammatory cytokines and chemokines, as well as changes in diverse immune cell types, indicative of hyperactive pro-inflammatory cellular states [4]. Immune dysfunction in Down syndrome has been attributed to the triplication of several important immunoregulatory genes. For example, 4 of the 6 interferon (IFN) receptors (*IFNAR1*, *IFNAR2*, *IFNGR2*, *IL10RB*) and several downstream target genes of IFN signaling (e.g., *MX1* and *MX*2) are clustered on chromosome 21 and play important roles in immunomodulation and viral pathogenesis [4,5,6,7]. Furthermore, transmembrane protease serine 2 (TMPRSS2), which cleaves the spike protein of SARS-CoV-2 and facilitates viral entry [8], is also located on chromosome 21 [9,10]. Most recently, it has been reported that *DYRK1A*, which is triplicated in Down syndrome, can upregulate angiotensin-converting enzyme 2 (ACE2), the main receptor for SARS-CoV-2 [11]. These clinical and molecular lines of evidence raise the possibility that individuals with Down syndrome are more susceptible to SARS-CoV-2 infection and can develop more severe COVID-19 [3].

A recent international survey conducted by the Trisomy 21 Research Society and the United Kingdom International Severe Acute Respiratory Infection Comprehensive Clinical Characterization Collaboration Consortium (UK ISARIC4C) indicated that COVID-19 patients with Down syndrome, especially those older than 40 years, are more vulnerable and are at higher risk for hospitalization and death [12]. Another cohort study of 8 million adults in the UK estimated a 4-fold increased risk for COVID-19-related hospitalization and a 10-fold increased risk for COVID-19-related death in individuals with Down syndrome [13]. However, because these epidemiological studies did not differentiate between differences in immune status, physiological/developmental differences, or social and behavioral factors that can greatly affect exposure to the virus and, thus, the severity of COVID-19, further experimental research is needed to clarify the drivers of worse health outcomes in Down syndrome.

We hypothesized that due to triplication of key genetic loci, people with Down syndrome would exhibit increased disease incidence and severity due to dramatically increased susceptibility and pro-inflammatory responses to infection. To test this hypothesis, we carried out experimental infections with SARS-CoV-2 in a mouse model of Down syndrome [14]. As predicted, mice with Down syndrome exhibited more severe clinical signs of illness as compared with non-Down syndrome littermates. We further undertook a series of studies by analyzing virus replication and histopathological changes in multiple organs, and local immune responses and genome-wide gene expression profiles in the lungs. While stronger innate immune responses might partially contribute to more severe clinical symptoms and lung histopathology in mice with Down syndrome, the results from molecular analyses suggest that other physiological and/or developmental processes, such as deficient oxidative phosphorylation and dysfunctions in cilium movement and the cardiopulmonary axis, may contribute to higher mortality in mice with Down syndrome, and should be considered in evaluating the pathogenesis of this disease. Our study represents the first effort to investigate SARS-CoV-2 infection in detail in a murine model of Down syndrome.

## 2. Materials and Methods

**Study design.** To ensure that the observed differences are attributable to Down syndrome-specific genetic or physiological factors rather than other variables, Down syndrome and non-Down syndrome mice were matched by age and sex. Both groups of mice were infected with the same number of infectious SARS-CoV-2 and maintained under the same environmental conditions, including the same number of mice per cage and the same distance between cages such that the potential virus transmission between animals within a cage or between cages would be similar.

**Cells and the virus.** Vero cells were cultured in Dulbecco’s modified Eagle’s medium (DMEM) (Gibco) containing 10% fetal bovine serum (FBS) and 1% penicillin and streptomycin (PS) at 37 °C in 5% CO_2_. SARS-CoV-2 strain USA-WA1/2020 was obtained through BEI Resources, the National Institute of Allergy and Infectious Diseases (NIAID), and the National Institutes of Health (NIH), and the virus was propagated in Vero cells. Virus titer was determined by standard virus plaque assays on Vero cells and expressed as plaque forming units per milliliter (PFU/mL). All experiments with live SARS-CoV-2 were performed in the BSL-3 facility at the University of Arkansas for Medical Sciences (UAMS) and approved by the Institutional Biosafety Committee.

**Mice.** B6.Cg-Tg(K18-ACE2)2Prlmn/J (Strain #:034860) (abbreviated as ACE2) mice were purchased from the Jackson Laboratory. Dp(16)1Yey/+ (abbreviated as Dp16) and ACE2 mice were both maintained in the C57BL/6J background. ACE2 mice were mated with Dp16 mice to produce Dp16/+;ACE2/+ (abbreviated as Dp16;ACE2) and ACE2/+ (abbreviated as ACE2) littermates. All mice were genotyped through PCR-based genotyping strategies. Dp16;ACE2 mice (n = 50) were identified by using the forward primer 5′-CTG CCA GCC ACT CTA GCT CT-3′ and the reverse primer 5′-AAT TTC TGT GGG GCA AAA TG-3′ as previously described [15], in addition to ACE2 allele. ACE2 mice (n = 53) were identified using 3 primers, namely 53,437 (5′-GAC CCC TGA GGG TTTC ATA TAG-3′), 53438 (5′-CAC CAA CAC AGT TTC CCA AC-3′), and 53439 (5′-AAG TTG GAG AAG ATG CTG AAA GA-3′), based on the protocol from the Jackson Laboratory.

All mice were maintained in a temperature- and moisture-controlled facility with a 12 h light–dark cycle from 6 AM–6 PM, and mice were given ad libitum access to food and water. All experimental procedures were approved by the Institutional Animal Care and Use Committees at UAMS and the Roswell Park Comprehensive Cancer Center.

**Animal infections.** All animal infection experiments were conducted in the animal biosafety level 3 (ABSL-3) facility with approval from the UAMS institutional biosafety committee and institutional animal ethics committee. Dp16;ACE2 and ACE2 mice of both sexes aged 8–18 weeks were housed in cages with high-efficiency particulate air filters within biosafety cabinets. Animal housing temperatures were maintained at 25 to 26 °C with 40% humidity. Mice were anesthetized with 50–100 mg/kg ketamine and 5–10 mg/kg xylazine delivered via intraperitoneal injection (IP) in a volume of 100 µL. A total of 40 Dp16;ACE2 mice and 43 ACE2 mice were then intranasally inoculated using a pipette with 20–40 µL medium containing SARS-CoV-2 at a dose of 2.5 × 10^4^ PFU per animal; a total of 10 Dp16;ACE2 mice and 10 ACE2 mice were mock-infected with PBS as control. At the required time points, mice were sacrificed via IP injection of sodium pentobarbital (150 mg/kg of body weight). Tissues were harvested for various measurements as described below.

**Assessment of clinical parameters and pulmonary function.** Mice were weighed and observed daily for any sign of illness. A score from 0 to 3 was assigned to each of the 3 clinical parameters (grooming/hunched, lethargy, response to stimulation): 0, normal; 1, slight; 2, moderate; 3, severe. An average score of the 3 parameters represents the clinical score for each mouse. Infected mice that exhibited severe disease or weight loss of ≥20% were humanely euthanized.

To evaluate pulmonary function, mice were analyzed by plethysmography. Mice were acclimated to a whole-body plethysmography chamber (1 mouse per chamber) for 15 min per day for 5 days prior to the onset of the experiment. During the experiment, mice were acclimated in the chamber for 15 min, followed by a 5 min data collection period. Plethysmography is non-invasive and does not involve any kind of restraint; it simply involves keeping the mouse in a chamber for a set time where the mouse is allowed to move freely. Respiration rate and other parameters were measured by analyzing air displacement in the chamber during continuous airflow. Animals were introduced into the chamber once per day, with their first introduction into the plethysmography chamber immediately prior to infection, and thereafter at a certain time point daily postinfection. The plethysmography chamber was cleaned with soap water after each use.

**Measurement of virus burden in the tissues.** Half of each organ was placed in 1 mL of PBS and homogenized using a tissue tearer. Homogenates were centrifuged at 10,000× *g*, and supernatants were collected and stored at −80 °C for measurement of virus titers or cytokines and chemokines. Pellets were resuspended in 1 mL TRIzol reagent (Invitrogen, Waltham, MA, USA), and total RNA samples were isolated as per the manufacturer’s instructions. Isolated RNA samples were treated with DNase at room temperature for 10 min to remove DNA contaminants, purified with an RNeasy mini spin column kit (Qiagen, Hilden, Germany, Cat#74104), and quantified with a NanoDrop 2000c spectrophotometer (Thermo Scientific, Waltham, MA, USA). Quantitative RT-PCR was carried out according to the manufacturer’s instruction (BioRad, Hercules, CA, USA). Briefly, for cDNA synthesis, 1 µg of each RNA sample was used for RT with iScript RT Supermix (BioRad cDNA kit, cart#1708841) or iScript NO-RT control Supermix for the negative control. The RT reaction was carried out in a thermal cycler (BioRad) at 25 °C for 5 min and at 46 °C for 20 min, and it was terminated at 95 °C for 1 min. PCR was carried out with an iTaq Universal SYBR Green Supermix kit (BioRad cat#1725121) in the MicroAmp optical 96-well plate (Applied Biosystems, Waltham, MA, USA, cat#N8010560) in a thermal cycler (QuantStudio 6 Flex, Applied BioSystems by Thermo Fisher Scientific, Waltham, MA, USA). The primer pair specific to the SARS-CoV-2 N gene (forward primer: 5′-ATG CTG CAA TCG TGC TAC AA-3′; reverse primer: 5′-GAC TGC CGC CTC TGC TC-3′) or to the actin gene (forward primer: 5′-ATG GAG GGG AAT ACA GCC C-3′; reverse primer: 5′-TTC TTT GCA GCVT CCT TCG TT-3′) were used for amplifying viral and cellular RNA, respectively. The amount of viral RNA in each sample was normalized to that of actin and expressed as relative to mock-infected samples.

**Histopathology.** Mouse tissues were fixed with 10% formalin for 30 min at room temperature and then processed, embedded with paraffin, and sectioned and stained with H&E at the Experimental Pathology Core facility at UAMS. The histopathology of the tissues was assessed blindly under a microscope. Images were taken under a microscope (Nikon, Tokyo, Japan) with an attached digital camera using SPOT 5 software.

**Flow cytometry analysis of immune cell infiltrates.** Mouse lungs were digested in collagenase solution (1.5 mg/mL collagenase type IV, 0.4 mg/mL DNase1, 10 mM HEPES, and 5% fetal bovine serum (FBS) in Hanks’ balanced salt solution) for 1 h. Tissue was mechanically sheared using dissection scissors, and the suspension was passed through a 70 µm mesh filter. Cells were centrifuged at 500× *g* for 5 min, washed once, and resuspended in 1x red blood cell (RBC) lysis buffer for 5 min. The suspension was diluted to 10 mL with PBS before being centrifuged again. Cells were resuspended in 1 mL of live/dead fixable aqua dead cell stain diluted in 1x PBS for 30 min at room temperature before being stained in PBS with 3% FBS (3% FBS/PBS) containing the following cell surface markers (1:500 dilution) for 30 min at 4 °C: CD45-phycoerthrin (clone 30-F-11, BD Biosciences, Milpitas, CA, USA), CD11b-Alexa Fluor 700 (clone M1/70, BD Biosciences), CD11c-brilliant violet 786 (clone HL3, BD Biosciences), F4/80-allophycocyanin (clone BM8, Invitrogen), and Ly6G-phycoerthrin-Cy7 (clone 1A8, BD Biosciences). Following staining, cells were centrifuged as described previously and fixed in 300 µL of 2% formalin in PBS for 15 min at room temperature prior to removal from the biosafety level 3 (BSL3) facility. Stained cells were analyzed based on fluorescence staining patterns to identify alveolar macrophages (F4/801CD11b^mid/low^CD11c^high^), CD11b^high^ interstitial/exudate macrophages (F4/801CD11b^high^CD11c^low/mid^), monocytes (F4/80-CD11b^high^CD11c^low^Ly-6G^−^), CD11c^high^ and CD11b^high or low^ dendritic cells (F4/80–CD11c^high^CD11b^high or low^), and neutrophils (F4/80–CD11c^low^CD11b^high^Ly-6G^+^). Data were analyzed with FlowJo software v10.8.1.

**Measurement of cytokines and chemokines.** Supernatants of the lung homogenates were collected and stored at −80 °C until use. To inactivate SARS-CoV-2 prior to removing the samples from the BSL-3 laboratory for cytokine assay, supernatants were placed at a distance of 14 cm from the ultraviolet (UV) light in a UV cross-linker (Fisher Scientific, Waltham, MA, USA) and exposed to UV light at an energy level of 1200 µW/ms for 15 min. Virus inactivation was confirmed by the absence of a cytopathic effect on Vero cells following infection with the inactivated samples and the absence of viral nucleoprotein by immunofluorescence assay. Homogenates were analyzed for cytokines and chemokines by Eve Technologies using their Mouse Cytokine/Chemokine 44-Plex Discovery Assay^TM^ Array (MD44) (www.evetechnologies.com (accessed on 2 December 2021)).

**Gene expression profiling by RNA-seq and differential expression analysis.** For RNA-seq analysis, RNAs were extracted from mouse tissues with TRIzol reagent (Invitrogen) and quantified with NanoDrop (Thermo Fisher, Waltham, MA, USA). The RNA samples were checked for quality using a Bioanalyzer (Agilent 2100) prior to RNA-seq analysis per the Novogene protocol (www.novogene.com (accessed on 6 December 2021)). The samples were sequenced and analyzed by Novogene. Differential expression analysis was conducted using the DESeq2 package (v1.40.2) [16] on the read counts data for ACE2 and Dp16;ACE2. Comparisons were made between the time points for each genotype. The *p* values were calculated using the Wald test, and values were corrected using the Benjamini and Hochberg method [17]. Genes with an adjusted *p* value smaller than 0.05 and a log fold change greater than 1 were considered significantly differentially regulated in the compared groups.

To examine the functional characteristics of the differentially regulated genes, gene set enrichment analysis (GSEA) was performed using the clusterProfiler package (v4.8.2) [18,19] with gene ontology (GO) annotations. The heatmaps were generated using the pheatmap package (v1.0.12) [20] and the ComplexHeatmap package (v2.16.0) [21]. The cnet plots were constructed using the enrichplot package (v1.20.0) [22].

**Statistical analysis.** Statistical analyses on cytokine and chemokine data were performed using Tukey’s multiple comparisons tests in the GraphPad Prism 9 program (v9.5.0). Other statistical analyses were carried out with unpaired *t*-tests or one-way ANOVAs in the same Prism 9 program. Results with *p* values of >0.05, <0.05, <0.01, <0.001, and <0.0001 are indicated in the legends. Cluster analysis was performed on gene expression profiling by RNA-seq analysis from 3 RNA samples isolated from the lungs of ACE2 or Dp16;ACE2 mice that were either mock-infected or infected with SARS-CoV-2.

## 3. Results

### 3.1. More Severe Clinical Signs of Illness and Higher Mortality Were Observed in the Mouse Model of Down Syndrome following Infection with SARS-CoV-2

Because many social and behavioral factors, such as maintaining distance from others and wearing masks, can greatly alter SARS-CoV-2 transmission and the outcome of COVID-19, to better understand COVID-19 pathogenesis it is critical that experimental infection be conducted to ensure that all conditions are under control. We chose to model SARS-CoV-2 infection in the Dp(16)1Yey/+ mouse mutant line, which is triplicated for the entire human chromosome 21 syntenic region on mouse chromosome 16 and currently the most widely used mouse model of Down syndrome [14,23,24,25,26,27]. At the time the study commenced, it was reported in a preprint that a transgenic mouse model that expresses human ACE2 receptor (K18-hACE2) was susceptible to SARS-CoV-2 infection that resulted in severe lung inflammation and impaired function [28,29]. Thus, Dp(16)1Yey/+ (abbreviated as Dp16) mice were mated with B6.Cg-Tg(K18-ACE2)2Prlmn/J mice to produce Dp16/+;ACE2/+ (abbreviated as Dp16;ACE2) and ACE2/+ littermates (abbreviated as ACE2). We then infected both Dp16;ACE2 and ACE2 mice intranasally with SARS-CoV-2 at 2.5 × 10^4^ plaque forming units (PFU) per mouse [29] and evaluated signs of illness including clinical scores (groomed/hunched, lethargy, response to stimuli), weight loss, mortality, and pulmonary function by plethysmography. In our initial study, we observed that 60% of Dp16;ACE2 mice died by day 6 p.i. (Supplementary Figure S1) and predicted further death if the experiment was extended. To generate the most data from this limited resource, we decided to sacrifice mice on day 6 p.i. for a detailed analysis of infection.

**Figure 1:**
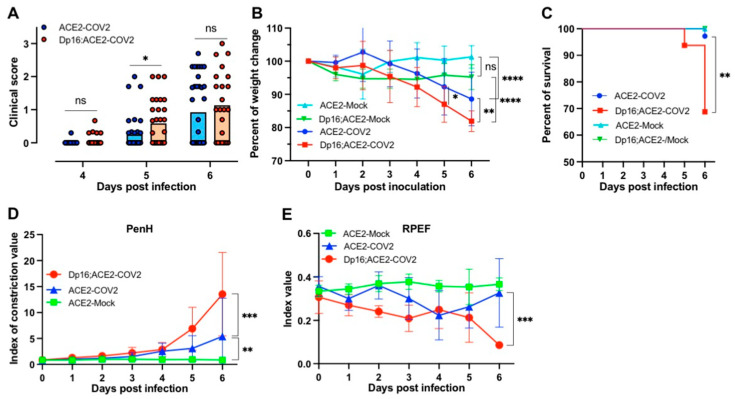
Clinical signs of COVID-19 in a mouse model of Down syndrome. ACE2 and Dp16;ACE2 mice were mock-infected (mock) or infected intranasally with SARS-CoV-2 (COV2) at 2.5 × 10^4^ PFU per mouse. (**A**) Clinical scores for infected mice. The scores are an average of 3 parameters (grooming/hunched, lethargy, and response to stimulation). Each parameter was scored from 0 to 3: 0, normal; 1, slight; 2, moderate; 3, severe. ACE2-COV2: n = 43, 36, and 35 at 4, 5, and 6 dpi, respectively. Dp16;ACE2-COV2: n = 40, 30, and 23 at 4, 5, and 6 dpi, respectively. Unpaired *t*-test, * *p =* 0.0253. Note that clinical scores for mock-infected mice remained at zero for both strains during days 4, 5, and 6. (**B**) Percent of weight change. n = 19 for ACE2-COV2; n = 38 for Dp16;ACE2-COV2; n = 10 for ACE2-Mock; n = 10 for Dp16;ACE2-Mock. ns, not significant; *, *p* = 0.0171; **, *p* = 0.0012; ****, *p* < 0.0001. (**C**) Percentage of survival. n = 36 for ACE2-COV2; n = 32 for Dp16;ACE2-COV2; n = 10 for ACE2-Mock; n = 6 for Dp16;ACE2-Mock. **, *p* = 0.0017 (log-rank (Mantel–Cox) test). (**D**,**E**) Two parameters (PenH and RPEF) for evaluating pulmonary functions by plethysmography (n = 4 for ACE2-Mock, n = 8 for ACE2-COV2, and n = 5 for Dp16;ACE2-COV2). ** and *** in (**D**) are *p* = 0.009 and 0.0001, respectively. *** in (**E**) indicates that *p* = 0.0009. ns, not significant.

As shown in Figure 1A, a small number of mice (1/43 for ACE2 and 5/40 for Dp16;ACE2) began to show clinical signs on day 4 postinfection (p.i.), and the disease progressed rapidly thereafter. Clinical signs of illness were generally more severe in Dp16;ACE2 mice than in ACE2 mice (*p* = 0.0253 on day 5 p.i.); the nonsignificant difference on day 6 p.i. might result from exclusion of the 10 dead mice. Similarly, the average weight loss was greater in Dp16;ACE2 mice (18.1%) compared to ACE2 mice (11.4%) by day 6 p.i. (*p* = 0.0012) (Figure 1B). The rapid progression of the disease between days 4 and 6 p.i. was particularly striking in Dp16;ACE2 mice. By day 5 p.i., 2 Dp16;ACE2 mice (6.25%) had succumbed to the disease, and by day 6 p.i., 10 Dp16;ACE2 mice (31.3%) had died, whereas only 1 ACE2 mouse (1/36, 2.8%) was deceased on day 6 p.i. (*p* = 0.0017) (Figure 1C). Pulmonary functions were evaluated in live mice daily by whole-body plethysmography. There are 15 different parameters that can be measured by plethysmography; here we include the results from two specific parameters (PenH and RPEF), as these parameters have been described previously for measuring respiratory function and have shown an excellent correlation with clinical symptoms for SARS-CoV-1 infection in mice [30]. PenH (enhanced pause) is a dimensionless index used to evaluate changes in the shape of the airflow pattern entering and leaving a plethysmograph as an animal breathes. Consistent with PenH values for SARS-CoV-1-infected mice [30], SARS-CoV-2-infected mice exhibited increased PenH values starting from day 4 p.i. (Figure 1D). Interestingly, SARS-CoV-2-infected Dp16;ACE2 mice exhibited even higher PenH values than ACE2 mice on day 6 p.i. (*p* = 0.0001) (Figure 1D). The index values of RPEF (ratio of time to peak expiratory flow relative to total expiratory time) for SARS-CoV-2-infected mice were generally lower than those for mock-infected control mice (Figure 1E). This result is also consistent with those previously reported for SARS-CoV-1-infected mice [30]. Furthermore, the Dp16;ACE2 mice had even lower RPEF values than ACE2 mice following SARS-CoV-2 infection, especially on day 6 p.i. (*p* = 0.0009) (compare Dp16;ACE2-COV2 (red) with ACE2-COV2 (blue) in Figure 1E). Thus, we concluded that mice with Down syndrome exhibited more severe disease than the ACE2 littermates following SARS-CoV-2 infection.

### 3.2. SARS-CoV-2 Replication Kinetics and Dissemination Were Similar between Mice with and without Down Syndrome

**Figure 2:**
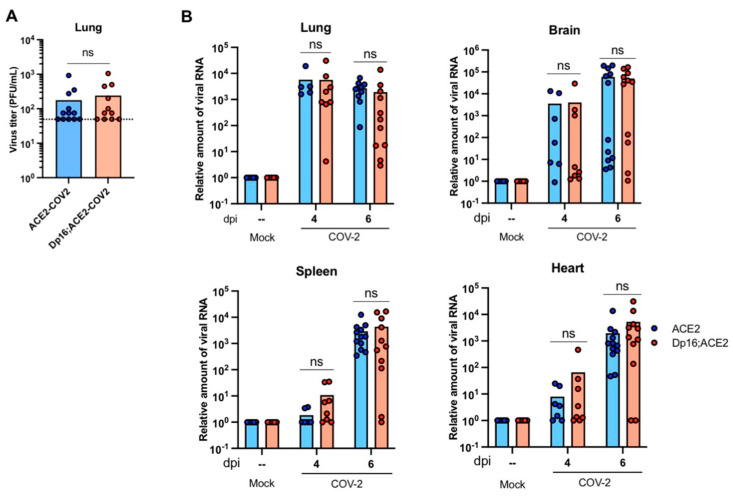
SARS-CoV-2 replication in a mouse model of Down syndrome. ACE2 and Dp16;ACE2 mice were mock-infected or infected intranasally with SARS-CoV-2 (COV2) at 2.5 × 10^4^ PFU per mouse. (**A**) Virus replication in the lungs. Virus titer in the lung homogenates at 4 dpi was determined by a plaque assay and expressed as PFU/mL. n = 12 for ACE2 mice; n = 11 for Dp16;ACE2 mice. The dash line indicates the detection limit. (**B**) Virus RNA in various mouse organs. Virus RNAs were isolated from lung, brain, spleen, and heart at 4 and 6 dpi and samples were quantified by qRT-PCR. Data are expressed as relative amount of viral RNA. ns, not significant.

Virus load in tissues reflects the efficiency of viral infection, replication, and dissemination in vivo and is often an important indicator for disease susceptibility and severity. Due to the increased severity of disease, we hypothesized that Dp16;ACE2 mice were more susceptible to infection. Thus, virus titers in lung homogenates were determined by plaque assays while viral RNAs in tissues were quantified by quantitative reverse transcription PCR (qRT-PCR) with primers specific to the SARS-CoV-2 nucleocapsid (N) gene. The results indicate that virus titers in the lungs were not significantly different between Dp16;ACE2 and ACE2 mice on day 4 p.i. (Figure 2A), which was consistent with viral RNA levels (Figure 2B). A dynamic virus dissemination process from the primary replication site (i.e., respiratory tract) to the secondary organs (i.e., brain, spleen, and heart) was revealed as evidenced by a decrease in viral RNAs in the lungs and a concomitant increase in the brain, spleen, and heart from day 4 to day 6 p.i. (Figure 2B). However, no significant difference in viral RNA levels was found between Dp16;ACE2 and ACE2 mice on both day 4 and day 6 p.i. in the four organs examined (*p* > 0.05) (Figure 2B), thus indicating that virus load might not be a major factor contributing to the more severe clinical disease observed in Dp16;ACE2 mice. Hence, any differences in disease progression between Dp16;ACE2 and ACE2 mice appear to be independent of viral replication and dissemination, and these can solely be attributed to the host response to infection.

### 3.3. Histopathological Analysis of the Lungs Infected with SARS-CoV-2 in the Mice with and without Down Syndrome

**Figure 3:**
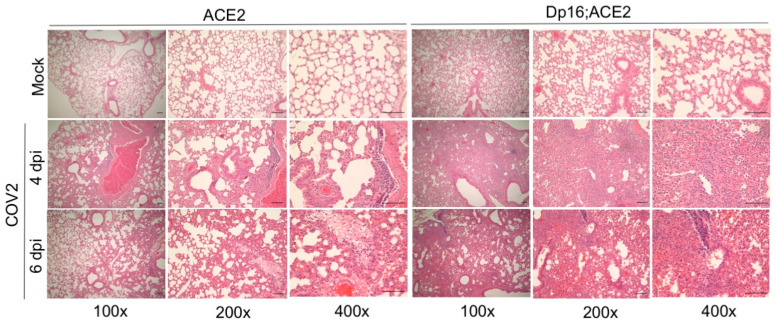
Histopathological analysis of the lungs infected with SARS-CoV-2 in ACE2 and Dp16;ACE2 mice. ACE2 and Dp16;ACE2 mice (n = 4–5 per group) were mock-infected (Mock) or intranasally infected with SARS-CoV-2 (COV2) at 2.5 × 10^4^ PFU. On 4 and 6 dpi, lungs were harvested, processed, and stained with hematoxylin and eosin (H&E). Severity of the lung histopathology was evaluated based on perivascular infiltrate, perivascular and focal collection into adjacent alveolar spaces with wall thickening, and infiltrate throughout the lung in alveolar and interstitial spaces. Representative images of the lung sections are shown at low (100x), medium (200x), and high (400x) magnification (scale bars, 50 µm).

We then examined the histopathological changes in the primary and secondary organs following intranasal infection with SARS-CoV-2 on days 4 and 6 p.i. While no obvious histopathological manifestation was observed in the brain, spleen, and heart, we found moderate perivascular infiltration of leukocytes and focal collection into adjacent alveolar spaces with wall thickening to severe leukocyte infiltration throughout the lungs in alveolar and interstitial spaces in both Dp16;ACE2 and ACE2 mice on days 4 and 6 p.i. (Figure 3). The extent of leukocyte infiltration in the lungs was more pronounced in Dp16;ACE2 mice than in ACE2 mice, as revealed by hematoxylin and eosin (H&E) staining of representative lung sections (Figure 3). Thus, the histopathological changes in the lungs were more pronounced and extensive in mice with Down syndrome and they were correlated with increased disease severity.

### 3.4. Differential Immune Responses to SARS-CoV-2 Infection in the Lungs between the Mice with and without Down Syndrome

**Figure 4:**
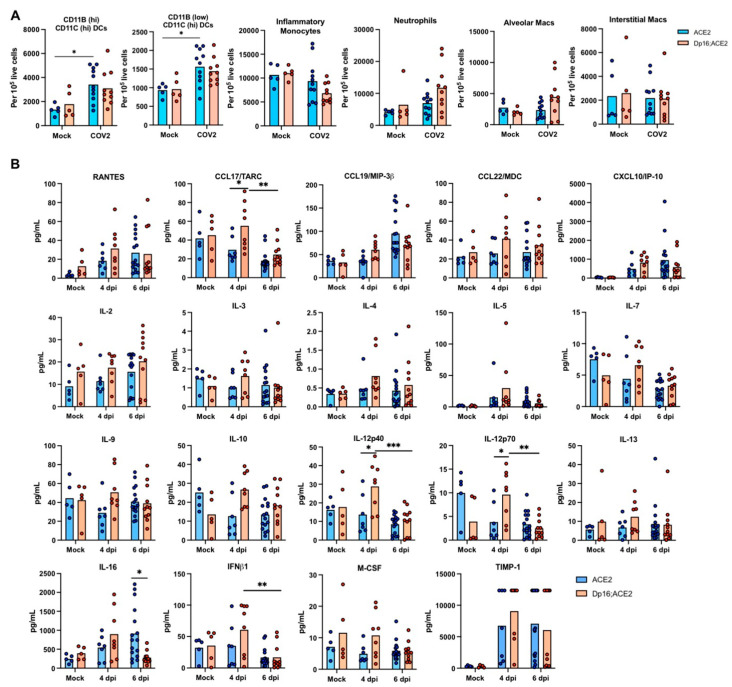
Immune response to SARS-CoV-2 infection in the lungs of ACE2 and Dp16;ACE2 mice. (**A**) Flow cytometric analysis of immune cells in the lungs from ACE2 and Dp16;ACE2 mice following mock infection (Mock) or intranasal infection with SARS-CoV-2 (COV2) at 2.5 × 10^4^ PFU at 6 dpi (2 independent experiments; n = 5 and 11 for mock and 6 dpi, respectively, for ACE2 mice; n = 5 and 10 for mock and 6 dpi, respectively, for Dp16;ACE2 mice). DCs, dendritic cells; Macs, macrophages. (**B**) Quantification of cytokine and chemokine proteins in lung homogenates from ACE2 and Dp16;ACE2 mice following mock infection (Mock) or intranasal infection with SARS-CoV-2 (COV2) at 2.5 × 10^4^ PFU at 4 and 6 dpi (4 independent experiments; n = 5, 7, and 16 for mock, 4 dpi, and 6 dpi, respectively, for ACE2 mice; n = 5, 8, and 11 for mock, 4 dpi, and 6 dpi, respectively, for Dp16;ACE2 mice). *, *p* < 0.05; **, *p* < 0.01; ***, *p* < 0.001 (two-way ANOVA).

As initial immune responses to SARS-CoV-2 infection in the lungs play a pivotal role in the early development and severity of COVID-19 in patients [31,32], we hypothesized that the increase in disease severity may be due to a more pronounced pro-inflammatory response to infection, and that Dp16;ACE2 mice may exhibit increases in neutrophil infiltration and pro-inflammatory cytokine induction compared with ACE2 mice. To test this hypothesis, we evaluated immune cell repertoire and a panel of 44 diverse cytokines and chemokines in the lungs of Dp16;ACE2 and ACE2 mice following infection with SARS-CoV-2. To our surprise, we observed no significant difference for the various types of leukocytes between Dp16;ACE2 and ACE2 mice (Figure 4A). Upon extensive analysis of the cytokine/chemokine expression profiles, we observed significantly increased expression of CCL17 and IL-12 on day 4 p.i. in Dp16;ACE2 mice compared to ACE2 mice (*p* < 0.05) (Figure 4B). However, for the vast majority of the cytokines/chemokines, the difference between Dp16;ACE2 and ACE2 mice was subtle (Figure 4B and Supplementary Figure S2). These results indicate that while only a small number of cytokines/chemokines were elevated in Dp16;ACE2 mice at the statistically significant level, a clear trend exists in which a large number of pro-inflammatory cytokines and chemokines were detected in the lungs at higher levels 4 days p.i. in Down syndrome mice.

### 3.5. Differential Gene Expressions in Response to SARS-CoV-2 Infection in the Lungs between the Mice with and without Down Syndrome as Revealed by RNA Sequencing Analysis

**Figure 5:**
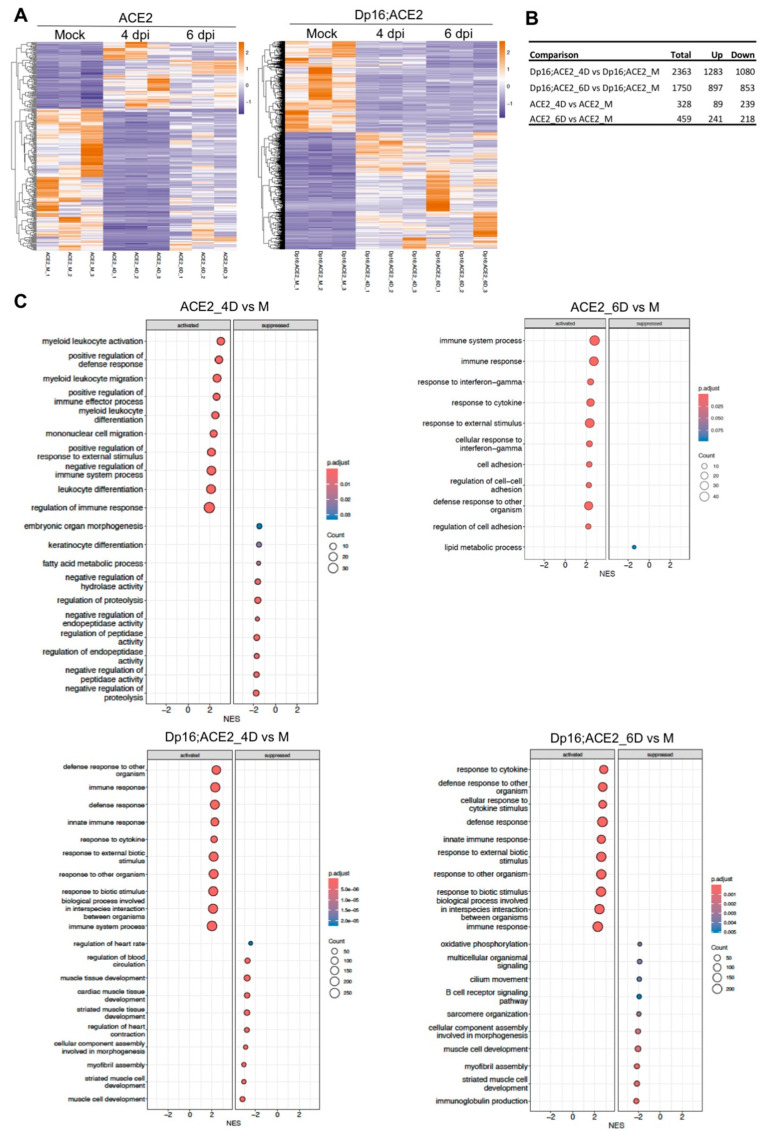

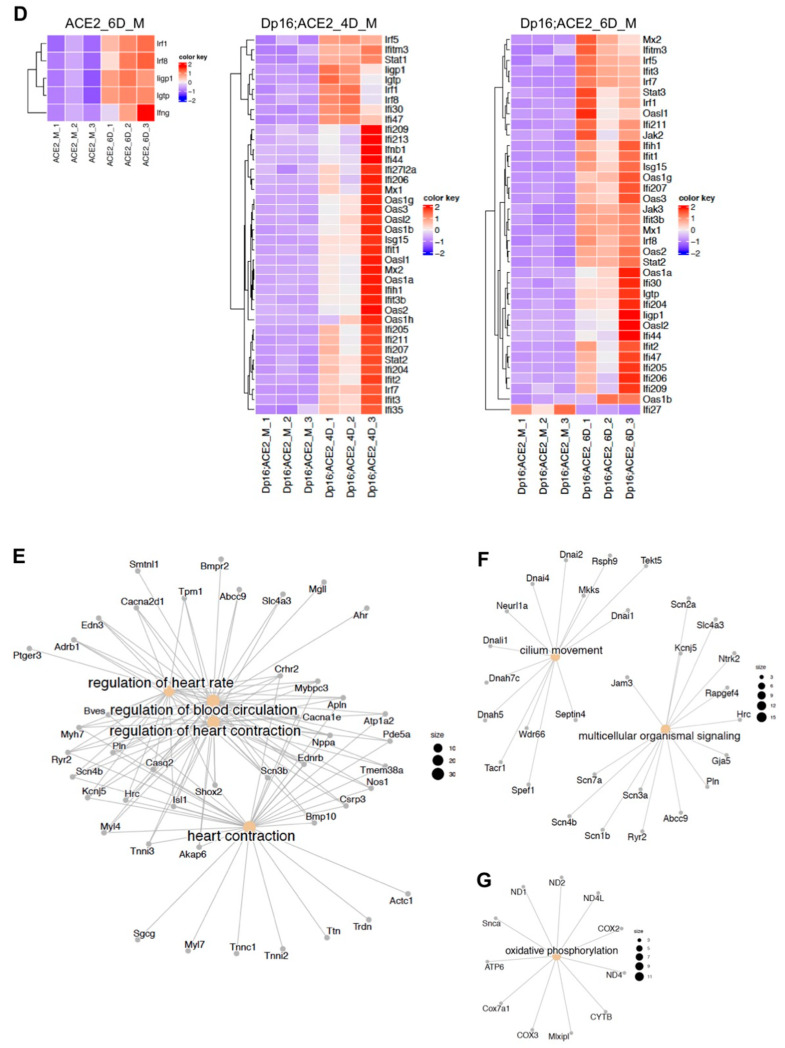
Gene expression profiling by RNA-seq analysis. Three RNA samples for each group were isolated from the lungs of ACE2 or Dp16;ACE2 mice that were either mock-infected (Mock) or infected with SARS-CoV-2; at 4 dpi or 6 dpi, they were subjected to RNA-seq analysis (n = 3). (**A**) A heatmap that illustrates the differentially expressed genes 4 dpi, 6 dpi, and Mock for the ACE2 and Dp16;ACE2 genotypes. (**B**) Summary of the number of differentially expressed genes between virus-infected samples and mock-infected samples. M represents mock-infected; 4D represents 4 dpi; 6D represents 6 dpi. (**C**) Top enriched terms (ranked by NES (normalized enrichment score)) from the pathway analysis results of the differentially expressed genes between 4 dpi (4D), 6 dpi (6D) and mock (M) for ACE2 and Dp16;ACE2 genotypes. The analysis was performed using the clusterProfiler package v. 4.8.3. (**D**) Heatmap showing the relative expression of IFN-related genes in virus-infected vs. mock-infected samples. The expression data was scaled by Z-score for the construction of the heatmaps. (**E**) Genes associated with the terms “regulation of heart rate (GO:0002027)”, “regulation of blood circulation (GO:1903522)”, “regulation of heart contraction (GO:0008016)”, and “heart contraction (GO:0060047)”, which are significantly enriched in downregulated genes at 4 dpi in Dp16;ACE2 samples. (**F**) Genes associated with the terms “cilium movement (GO:0003341)” and “multicellular organismal signaling (GO:0035637)”, which are significantly enriched in downregulated genes at 6 dpi in Dp16;ACE2 samples. (**G**) Genes associated with the term “oxidative phosphorylation (GO:0006119)”, which is significantly enriched in downregulated genes at 6 dpi in Dp16;ACE2 samples.

As described in the previous sections, we observed more severe disease, significantly increased mortality (Figure 1), and lung pathology (Figure 3) in Dp16;ACE2 mice. To seek the underlying clues beyond the limited differences in immune responses in the mouse mutants with or without Down syndrome, we carried out RNA-seq analysis. Thus, we infected both ACE2 and Dp16;ACE2 mice intranasally with SARS-CoV-2 or mock-infected as controls. On days 4 and 6 p.i., lungs were harvested and RNAs isolated for transcriptomic profiling. As shown in Figure 5A, SARS-CoV-2 infection profoundly altered the transcriptomic landscape in both ACE2 and Dp16;ACE2 mice, as illustrated by the color change in the heatmap from mock to 4 and 6 days p.i. The changes in the expression patterns were further divergent between infected ACE2 and Dp16;ACE2 mice. For example, in response to SARS-CoV-2 infection, the expression of 2363 genes (1283 genes up, 1080 genes down) on day 4 p.i. and of 1750 genes (897 genes up, 853 genes down) on day 6 p.i. in Dp16;ACE2 mice was altered (Figure 5B). In contrast, only 328 genes (89 genes up, 239 genes down) and 459 genes (241 genes up, 218 genes down) were differentially expressed on day 4 and day 6 p.i., respectively, in ACE2 mice following virus infection (Figure 5B). Thus, the transcriptional landscape between Dp16;ACE2 and ACE2 mice is distinct in response to SARS-CoV-2 infection.

We further performed bioinformatics analysis to identify networks and pathways that may drive the common and unique gene expression in ACE2 and Dp16;ACE2 mice. Figure 5C shows the top 10 pathways either upregulated (activated) or downregulated (suppressed) in ACE2 and Dp16;ACE2 mice in response to SARS-CoV-2 infection, which illuminates several major patterns, as described below.

First, all top 10 pathways that were upregulated in ACE2 and Dp16;ACE2 mice at both timepoints following SARS-CoV-2 infection were related to host immune and defense responses (Figure 5C). This finding is consistent with a more pronounced infiltration of leukocytes (Figure 3), elevation of dendritic cells, neutrophils, and alveolar macrophages (Figure 4A), and induction of pro-inflammatory cytokines and chemokines (Figure 4B) in the lungs in both Dp16;ACE2 and ACE2 mice following SARS-CoV-2 infection.

Second, when compared between ACE2 and Dp16;ACE2 mice, the same five top pathways were activated in ACE2 mice on day 6 p.i. and in Dp16;ACE2 mice on day 4 p.i., thus indicating that Dp16;ACE2 mice respond to virus infection more rapidly (Figure 5C). Similarly, IFN-related/stimulated genes were induced more significantly in both the number of genes and the level of induction in Dp16;ACE2 mice than in ACE2 mice (Figure 5D). In ACE2 mice, no IFN-related gene was significantly induced on day 4 p.i. and only 5 IFN-related genes were induced on day 6 p.i. (adj. *p* < 0.05) (Figure 5D). In contrast, more than 30 IFN-related genes were induced on both days 4 and 6 p.i. in Dp16;ACE2 mice, and only 1 gene (*Ifi27*) was suppressed on day 6 p.i. (Figure 5D). This result is consistent with the triplication of *Ifnar1*, *Ifnar2*, and *Ifngr2* in the Dp16;ACE2 mice [27] and indicates that mice with Down syndrome have a hyperactive IFN-I response during acute SARS-CoV-2 infection.

Third, the top 10 enriched pathways that were suppressed by SARS-CoV-2 infection on day 4 p.i. were unique in Dp16;ACE2 mice, and many were related to the cardiopulmonary structure and function, such as myofibril assembly and the regulation of blood circulation and heart contraction (Figure 5C). Of particular note is the clusters of over 40 interconnected genes that are involved in and regulate heart rate, heart contraction, and blood circulation (Figure 5E). This contrasts with infection in ACE2 mice, in which the top downregulated pathways are mainly involved in the regulation of peptidase and hydrolase activities, proteolysis, and the lipid metabolic process (Figure 5C). By 6 days p.i., many of the same pathways were continuously suppressed in Dp16;ACE2 mice, with 5 new pathways emerging to the top 10: cilium movement (14 genes), multicellular organismal signaling (15 genes), oxidative phosphorylation (11 genes), B cell receptor signaling, and immunoglobulin production (Figure 5C,F,G). Suppression of B cell receptor signaling and immunoglobulin production could result in a weakened adaptive immune response to vaccination, a common deficiency found in people with Down syndrome [33]. These data support the possibility of a correlation between deficient oxidative phosphorylation coupled with cardiopulmonary dysfunction and the suppression of cilium movement and the high mortality occurred in Dp16;ACE2 mice on day 6 p.i. (Figure 1).

## 4. Discussion

### 4.1. Susceptibility of Individuals with Down Syndrome to SARS-CoV-2 Infection

To date, the question as to whether individuals with Down syndrome are more susceptible to SARS-CoV-2 infection remains controversial. On the one hand, clinical and epidemiological studies have shown that respiratory disease constitutes a large proportion of the morbidity in Down syndrome [34], and lung disease accounts for 54% of hospital admissions in Down syndrome [35]. Individuals with Down syndrome have an increased frequency of respiratory tract infection [36] and acute respiratory distress syndrome [37]. It has been reported that inherent dysregulations in innate and acquired immunity contribute to this predisposition to respiratory tract infection in Down syndrome, and the poor response to vaccination potentially contributes to respiratory infection [33,34]. Particularly relevant to SARS-CoV-2 infection is the genetic evidence that *TMPRSS2* is triplicated in Down syndrome [38]. TMPRSS2 is shown to be involved in SARS-CoV-2 entry into cells by priming the spike protein of SARS-CoV-2, and inhibition of TMPRSS2 by an inhibitor blocked SARS-CoV-2 spike protein-mediated entry into lung cells [8]. Thus, overexpression of TMPRSS2 in Down syndrome could potentially enhance susceptibility to SARS-CoV-2 infection. Another triplicated gene in Down syndrome, *DYRK1A*, has recently been shown to upregulate the ACE2 receptor in human cells [11]. Furthermore, SARS-CoV-2 enters cells via endocytosis. Dysregulation of endocytosis in Down syndrome has been linked to an increased dose of several endosomal pathway-related genes that map to chromosome 21, such as amyloid precursor protein (*APP*), synaptojanin-1 (*SYNJ1*), intersectin-1 (*ITSN1*), and regulator of calcineurin 1 (*RCAN1*) [23,39]. Together, these lines of evidence support the notion that individuals with Down syndrome might be more susceptible to SARS-CoV-2 infection.

On the other hand, studies have also pointed out an opposite outcome in Down syndrome during viral infection. For example, four IFN receptor genes (*IFNAR*1, IFNAR2, *IFNGR2*, and *IL10RB*) and two IFN-stimulated genes (*MX1* and *MX2*) are triplicated in Down syndrome, and individuals with Down syndrome exhibit hypersensitivity to type I IFN (IFN-I) [4,6]. As IFN-Is are pro-inflammatory cytokines that also have potent antiviral activity against diverse viruses, overactivation of IFN-I signaling pathways in Down syndrome might lead to resistance to viral infections. Indeed, a recent epidemiological study of a large cohort of individuals with Down syndrome revealed protection from most infections in Down syndrome compared with non-Down syndrome controls, including influenza A virus, unspecified upper respiratory infections, mononucleosis, varicella zoster virus, and intestinal infections [40,41]. This evidence supports the notion that individuals with Down syndrome are less susceptible to initial viral infection.

Furthermore, information gained from epidemiological surveys on COVID-19 incidence has significant limitations for drawing conclusions on susceptibility to SARS-CoV-2 infection. This is because it is difficult to control many social and behavioral factors (such as social distancing, mask wearing, and personal hygiene) in these epidemiological studies. And yet, these factors can drastically affect how many virus particles an individual initially is infected with and, thus, the outcome of the infection. Thus, it remains unclear whether individuals with Down syndrome are more susceptible to SARS-CoV-2 infection and more severe COVID-19 disease.

We addressed this important question by employing the mouse model of Down syndrome that has been widely used in the Down syndrome research community [14,23,24,25,26,27] for experimental infection with SARS-CoV-2, such that all experimental parameters and conditions are under control. Following intranasal inoculation with the same number of infectious viruses, the susceptibility of individual mice to SARS-CoV-2 was assessed by determining the virus loads in various organs. Our data show that virus loads either in the primary organ (lung) or the secondary organs (brain, spleen, and heart) were similar between mice with and without Down syndrome (Figure 2), thus indicating that the two groups of mice have similar susceptibility to SARS-CoV-2 infection. One possible interpretation of this result is that the resistance (antiviral) arm may counteract the susceptibility arm of the triplicated genes in Down syndrome. Although more viruses possibly enter into cells during initial infection in the respiratory tract due to overexpression of TMPRSS2 and other susceptibility genes, hyper-responsiveness to IFN-I could potentially dampen subsequent viral replication in Dp16;ACE2 mice. As a result, there is no significant difference in susceptibility to SARS-CoV-2 infection between Dp16;ACE2 mice and their ACE2 littermates. It is worth noting that although DYRK1A can upregulate ACE2 expression in human cells, it has no effect on mouse ACE2 expression in conditionally *Dyrk1a*-knocked out mice, suggesting species-specificity of DYRK1A-mediated regulation of ACE2 [11]. This result is consistent with our RNA-seq data, which show that mouse ACE2 expression was unaffected, though DYRK1A was overexpressed in Dp16;ACE2 mice (data not shown). However, the level of human ACE2 expression in the lungs of K18-hACE2-transgenic mice declined over the course of SARS-CoV-2 infection [42]. Thus, it remains to be seen whether overexpression of DYRK1A in people with Down syndrome can overcome the downregulation of ACE2 by SARS-CoV-2 infection.

### 4.2. Mechanism of SARS-CoV-2 Pathogenesis in Individuals with Down Syndrome

While a plethora of evidence support the hypothesis that SARS-CoV-2 infection causes more severe disease in individuals with Down syndrome [3], the underlying mechanism remains unknown. There are multiple factors that may contribute to the severity of the disease in Down syndrome. These include anatomical defects in the respiratory tract (e.g., airway malacia, smaller trachea) that cause obstructive sleep apnea [43,44], congenital heart defects that result in cardiopulmonary dysfunction and pulmonary hypertension [45,46], dysregulation of innate and adaptive immunity that leads to inflammatory and autoimmune responses [4], and premature aging that may lead to Alzheimer disease and immunosenescence [2,47]. Consistent with this interpretation, our data show that Dp16;ACE2 mice exhibited more severe clinical symptoms and lung histopathology than ACE2 mice following SARS-CoV-2 infection (Figure 1 and Figure 3). In general, viral disease progression and severity often correlate with high viral replication (load) in a host for a given virus strain. However, in comparisons between Dp16;ACE2 mice and ACE2 littermates, there were no significant differences in the overall virus loads in the lungs and other organs (Figure 2). This suggests that other factors likely contributed to the more severe disease phenotype in Down syndrome.

Then, what drives the severity of the disease in Down syndrome? Since cytokine storm is the hallmark of COVID-19 severity in patients, we assessed a panel of 44 pro-inflammatory cytokines and chemokines in the lungs following virus infection. Our data show that while only a few cytokines/chemokines were statistically significantly elevated in Down syndrome mice, there was a clear trend in which a large number of pro-inflammatory cytokines and chemokines were secreted in the lungs at higher levels at an earlier time point after infection (4 days p.i.) in Down syndrome mice (Figure 4B). This finding indicates that an overall accelerated and elevated innate immune response may drive the severity of the disease in Down syndrome. This interpretation is also consistent with the data from the RNA-seq analysis, which show that Dp16;ACE2 mice had a more accelerated and exacerbated inflammatory immune response to SARS-CoV-2 infection than the ACE2 littermates (Figure 5C). Thus, dysregulation of innate immunity in Down syndrome likely contributes to the predisposition toward more rapid progression and more severe COVID-19.

Furthermore, overactivation of the IFN signaling pathways in Down syndrome may also contribute to the heightened disease severity in COVID-19. Consistent with this interpretation is our RNA-seq data, which show that Dp16;ACE2 mice had a significantly more robust response to IFN-I and IFN-II, both in terms of the number of IFN-stimulated genes and the number of mRNAs, than the ACE2 littermates (Figure 5D). Although both IFN-I and IFN-II have antiviral activities, these are also, and perhaps primarily, inflammatory cytokines and can lead to interferonopathies [48,49]. A recent in vitro study revealed that fibroblast cells derived from persons with Down syndrome exhibited a biphasic effect during the IFN-I response [41]. During the initial phase, hyper-response to IFN-I due to triplication of *IFNAR1* and *IFNAR2* sensitizes the cells to an antiviral state, resulting in reduced infection by influenza A virus. However, at the late phase, overactivation of the IFN-I signaling pathways in turn desensitizes the subsequent response to IFN-I, resulting in increased viral susceptibility. The authors attribute this phenomenon to a negative feedback mechanism. Their results indicate that individuals with Down syndrome have fewer viral infections, but, when infected, they suffer from more severe disease [41], which is consistent with our observations in the mouse model of Down syndrome. A recent genetic study using the mouse model of Down syndrome also shows that in addition to exacerbation of the immune responses, triplication of the *Ifnr* locus contributes to other abnormalities typically seen in Down syndrome, such as heart defects, developmental delays, and craniofacial abnormalities [27]. Thus, the exacerbated IFN-I and IFN-II responses to SARS-CoV-2 infection observed in Dp16;ACE2 mice (Figure 5D) may contribute to the more severe COVID-19 in Down syndrome.

### 4.3. Potential Causes for the High Mortality of COVID-19 in Down Syndrome

A large cohort study of 8 million adults, of which 4053 had Down syndrome, estimated that there is a 10-fold increased risk for COVID-19-related death in persons with Down syndrome [13]. In general agreement with these epidemiological findings, our data from the mouse model of Down syndrome show that the mortality rate for COVID-19 was ≈11-fold higher by day 6 p.i in Dp16;ACE2 mice (31.3%) than in ACE2 littermates (2.8%) (Figure 1C). However, it remains unknown what causes the higher mortality of COVD-19 in Down syndrome. We found that there are no overt clinical signs of severe illness prior to death for the mice with Down syndrome following SARS-CoV-2 infection. This observation raises an interesting but intriguing question as to what causes the high mortality. Confronted by this puzzle, we undertook molecular analysis on RNA samples from mouse lungs. On the basis of the RNA-seq data (Figure 5), we postulate that the following three interconnected molecular pathways or networks might contribute to the high mortality in Dp16;ACE2 mice following SARS-CoV-2 infection.

#### 4.3.1. Cardiopulmonary Dysfunction

It is well documented that heart and lung diseases are the leading causes of death for persons with Down syndrome [34]. Pneumonia and infectious lung disease, congenital heart defects, and circulatory disease account for approximately 75% of all deaths in persons with Down syndrome [34]. Interestingly, we found in our RNA-seq analysis that all top 10 enriched pathways on day 4 p.i. and 5 of the top 10 enriched pathways on day 6 p.i., which are related to regulation of the cardiopulmonary structure and function, especially the smooth muscle cells, were significantly suppressed in Dp16;ACE2 mice but not in ACE2 littermates following SARS-CoV-2 infection (Figure 5C). Over 40 genes are involved in the 4 interconnected pathways, namely regulation of heart rate, regulation of blood circulation, regulation of heart contraction, and heart contraction (Figure 5E). It is, thus, conceivable that suppression of the vast array of genes involved in the regulation of cardiopulmonary function may result in dysfunction and failure of the heart and lungs and consequently the high mortality rate.

#### 4.3.2. Deficient Mucociliary Clearance

In the airways, cilia function in concert with airway mucus to mediate the critical function of mucociliary clearance, cleansing the airways of inhaled particles and pathogens, thus maintaining the mucociliary escalator [50]. The mucociliary escalator is a layer of fluid and mucins that lines the epithelium and that cleans the airways by moving continuously from the lower respiratory tract cephalad. When there is dysfunction of the mucociliary escalator, the defenses of the epithelium are markedly weakened, resulting in lung disease [50]. Our RNA-seq analysis revealed that a cluster of 14 genes that regulate cilium movement are significantly suppressed following SARS-CoV-2 infection in Dp16;ACE2 mice (Figure 5C,F), which may contribute to deficient mucociliary clearance. It has been reported that although the ciliary ultrastructure is normal, ciliary beat frequency is decreased in people with Down syndrome [51], which could be further deteriorated by SARS-CoV-2 infection. A number of bacterial and viral pathogens have been reported to cause deficient mucociliary clearance, including *Actinobacillus pleuropneumoniae*, *Pseudomonas aeruginosa*, *Moraxella catarrhalis*, *Mycoplasma pneumoniae*, *Mycoplasma hyopneumoniae*, and *Bordetella species* that specifically target ciliated cells for adherence [52], as well as respiratory syncytial virus [53]. In addition, the host response to infection may contribute to deficient mucociliary clearance. An earlier study has shown that human neutrophil elastase causes epithelial disruption and, at high concentrations, reduces cilia beat frequency [54]. Additionally, reactive oxygen species generated by polymorphonuclear leukocytes, especially hydrogen peroxide, can decrease cilia beat frequency [55]. Thus, deficient mucociliary clearance combined with severe pneumonia as, evidenced by the widespread infiltration of leukocytes throughout the lungs (Figure 3), may have led to difficulty in breathing and contributed to the high mortality observed.

#### 4.3.3. Deficient Oxidative Phosphorylation

In our transcriptomic profiling analysis, we found that oxidative phosphorylation was one of the top enriched pathways that was suppressed in Dp16;ACE2 mice following SARS-CoV-2 infection on day 6 p.i. (Figure 5C), and this pathway was associated with a cluster of 11 genes (Figure 5G); these findings suggest that suppression of oxidative phosphorylation may contribute to COVID-19 mortality in Down syndrome. Oxidative phosphorylation is essential for the survival of higher animals, as oxidative phosphorylation not only provides most of the ATP used by higher animals to support life but is also responsible for setting and maintaining metabolic homeostasis [56]. Oxidative phosphorylation requires large amounts of oxidizable substrate and molecular oxygen. In higher animals, well-developed lungs, heart, and blood vessels work together to form a highly coordinated and responsive nutrient delivery (and waste removal) system. This delivery system can supply oxygen and remove waste products (notably CO_2_) at rates matched accurately to the metabolic requirements of individual tissues. To function properly, the cardiopulmonary–vascular system needs sufficient capacity to meet maximal demand. Oxidative phosphorylation has the greatest metabolite delivery requirement, and it is of central importance to regulation of the cardiopulmonary–vascular system [56]. Additionally, oxidative phosphorylation has been linked to regulation of the afferent neural activity associated with the carotid body blood. This afferent nerve activity travels to both the brain and the diaphragm, helping to regulate breathing and cardiac output in order to maintain the oxygen level in the carotid artery [56]. Tissues highly dependent on oxygen, such as the cardiac muscle, skeletal and smooth muscle, central and peripheral nervous system, kidney, and insulin-producing pancreatic β-cells, are especially susceptible to defective oxidative phosphorylation. Therefore, suppression of oxidative phosphorylation may result in cardiopulmonary dysfunction and deficient mucociliary clearance. However, it remains to be seen whether defective oxidative phosphorylation can directly cause downregulation of the expression of genes that regulate the functions of the highly oxygen-dependent cardiac muscle, vascular and pulmonary smooth muscles, and motility of cilia (Figure 5E,F). Furthermore, there is evidence that defective oxidative phosphorylation plays an important role in atherogenesis and in the pathogenesis of Alzheimer’s disease, Parkinson’s disease, diabetes, and aging. The increased *APP* gene dose due to its triplication in Down syndrome is the leading cause of Alzheimer’s disease in Down syndrome and is also associated with oxidative stress [47]. Thus, coupled with cardiopulmonary dysfunction and deficient mucociliary clearance, deficient oxidative phosphorylation could contribute to the high mortality of COVID-19 in Down syndrome.

### 4.4. Limitations and Future Studies

The rate of infection by SARS-CoV-2 and the outcome of COVID-19 disease in people, including those with Down syndrome, exhibit considerable variation and are influenced by many factors, such as age, sex, immune status, pre-existing medical conditions, and environmental, behavioral, and social determinants. Although we have endeavored to control for these variables in our experimental infection model, inherent limitations exist in evaluating the social and behavioral aspects, potentially influenced by genetic and physiological differences in Down syndrome mice. Nonetheless, our results indicating comparable viral loads across various organs in both Dp16;ACE2 and ACE2 mice suggest that the presumed social and behavioral differences in Dp16;ACE2 mice likely do not impact infection rates. While the Dp16 model is the currently most widely used animal model for studying the pathogenesis of Down syndrome, it may not perfectly replicate all human Down syndrome characteristics due to certain species-specific differences.

Although SARS-CoV-2 lung infection in K18-hACE2 transgenic mice provides a model for studying severe infection that recapitulates features of COVID-19 in humans [42], several limitations are noted. Chiefly, the expression of the human ACE2 transgene is non-physiological, such as on a different chromosome, under a different promoter, and at different levels in different organs. Additionally, since ACE2 can regulate cardiovascular function, one argument is that transgenic expression of hACE2 may sensitize the Down syndrome mice and cause high mortality following SARS-CoV-2 infection. However, hACE2 expression level is reportedly decreased in K18-hACE2 mice on day 2 p.i. and continuously until day 7 p.i. [42].

The observed difference in clinical signs of illness and molecular evidence between ACE2 mice and Dp16;ACE2 mice, which were generated using the same K18-hACE2 transgenic mice, warrants further investigation. Future studies using mouse-adapted SARS-CoV-2 mutants [57] in wild-type and Dp16 mice may clarify whether the high mortality observed in Dp16;ACE2 mice is unique to the K18-hACE2 transgene, due to Down syndrome, or is a result of the combination thereof. Identifying the pathways associated with the severity and high mortality rates in Down syndrome could open new research avenues, including the exploration of potential therapeutic interventions targeting these pathways. Additionally, the findings from this study may have wider implications for understanding Down syndrome as a comorbidity factor in other infectious diseases, especially those affecting the cardiopulmonary system, such as influenza and respiratory syncytial virus infections.

This study uncovered more severe responses in Down syndrome mice compared to non-Down syndrome control mice upon infection with SARS-CoV-2, thereby creating a novel opportunity to elucidate the mechanisms driving these differences for the first time. Our team and others have engineered a significant number of mouse mutants with smaller chromosomal duplications and deletions within the Dp16 triplicated region [58,59], which will enable systematic genetic dissection to identify the minimal critical genomic regions and the dosage-sensitive genes responsible for the varied responses observed in Dp16. The identification of these genes and their protein products will not only advance our understanding of the underlying mechanisms but will also provide rational targets for developing therapeutic strategies aimed at addressing the specific clinical manifestations of Down syndrome.

## 5. Conclusions

In this study, we have investigated whether individuals with Down syndrome are more vulnerable to COVID-19 and what potential factors contribute to the disease phenotype using a mouse model of Down syndrome. We found that mice with and without Down syndrome have similar susceptibility to SARS-CoV-2 infection. However, Down syndrome mice exhibit more severe disease and higher mortality. While dysregulation of the innate immune system may partially contribute to the more severe disease phenotype, molecular analysis suggests that deficient oxidative phosphorylation coupled with cardiopulmonary dysfunction and deficient mucociliary clearance might also play an important role in causing the heightened disease severity and mortality observed in Down syndrome mice following SARS-CoV-2 infection.

## Data Availability

All data supporting the findings of this study are located within the paper, and these data are available from the relevant corresponding authors upon request. RNA-seq datasets generated in this study are available from the Gene Expression Omnibus (accession code: GSE260583).

## Supplementary Materials

The following supporting information can be downloaded at: https://www.mdpi.com/article/10.3390/biomedicines12030543/s1, Figure S1: Percentage of survival of ACE2 and Dp16;ACE2 mice following SARS-CoV-2 infection; Figure S2. Additional chemokine and cytokine responses to SARS-CoV-2 infection in the lungs of ACE2 and Dp16;ACE2 mice.

## References

[1] Antonarakis S.E., Skotko B.G., Rafii M.S., Strydom A., Pape S.E., Bianchi D.W., Sherman S.L. (2020). Down syndrome. Nat. Rev. Dis. Primers.

[2] Gensous N., Bacalini M.G., Franceschi C., Garagnani P. (2020). Down syndrome, accelerated aging and immunosenescence. Semin. Immunopathol..

[3] Espinosa J.M. (2020). Down Syndrome and COVID-19: A Perfect Storm?. Cell Rep. Med..

[4] Waugh K.A., Araya P., Pandey A., Jordan K.R., Smith K.P., Granrath R.E., Khanal S., Butcher E.T., Estrada B.E., Rachubinski A.L. (2019). Mass Cytometry Reveals Global Immune Remodeling with Multi-lineage Hypersensitivity to Type I Interferon in Down Syndrome. Cell Rep..

[5] Araya P., Waugh K.A., Sullivan K.D., Núñez N.G., Roselli E., Smith K.P., Granrath R.E., Rachubinski A.L., Estrada B.E., Butcher E.T. (2019). Trisomy 21 dysregulates T cell lineages toward an autoimmunity-prone state associated with interferon hyperactivity. Proc. Natl. Acad. Sci. USA.

[6] Sullivan K.D., Lewis H.C., Hill A.A., Pandey A., Jackson L.P., Cabral J.M., Smith K.P., Liggett L.A., Gomez E.B., Galbraith M.D. (2016). Trisomy 21 consistently activates the interferon response. eLife.

[7] Pichlmair A., Reis e Sousa C. (2007). Innate recognition of viruses. Immunity.

[8] Hoffmann M., Kleine-Weber H., Schroeder S., Krüger N., Herrler T., Erichsen S., Schiergens T.S., Herrler G., Wu N.H., Nitsche A. (2020). SARS-CoV-2 Cell Entry Depends on ACE2 and TMPRSS2 and Is Blocked by a Clinically Proven Protease Inhibitor. Cell.

[9] Dierssen M. (2012). Down syndrome: The brain in trisomic mode. Nat. Rev. Neurosci..

[10] De Toma I., Dierssen M. (2021). Network analysis of Down syndrome and SARS-CoV-2 identifies risk and protective factors for COVID-19. Sci. Rep..

[11] Strine M.S., Cai W.L., Wei J., Alfajaro M.M., Filler R.B., Biering S.B., Sarnik S., Chow R.D., Patil A., Cervantes K.S. (2023). DYRK1A promotes viral entry of highly pathogenic human coronaviruses in a kinase-independent manner. PLoS Biol..

[12] Hüls A., Costa A.C., Dierssen M., Baksh R.A., Bargagna S., Baumer N.T., Brandão A.C., Carfi A., Carmona-Iragui M., Chicoine B.A. (2021). Medical vulnerability of individuals with Down syndrome to severe COVID-19–data from the Trisomy 21 Research Society and the UK ISARIC4C survey. EClinicalMedicine.

[13] Clift A.K., Coupland C.A., Keogh R.H., Hemingway H., Hippisley-Cox J. (2021). COVID-19 Mortality Risk in Down Syndrome: Results From a Cohort Study of 8 Million Adults. Ann. Intern. Med..

[14] Li Z., Yu T., Morishima M., Pao A., LaDuca J., Conroy J., Nowak N., Matsui S.-I., Shiraishi I., Yu Y.E. (2007). Duplication of the entire 22.9 Mb human chromosome 21 syntenic region on mouse chromosome 16 causes cardiovascular and gastrointestinal abnormalities. Hum. Mol. Genet..

[15] Jiang X., Liu C., Yu T., Zhang L., Meng K., Xing Z., Belichenko P.V., Kleschevnikov A.M., Pao A., Peresie J. (2015). Genetic dissection of the Down syndrome critical region. Hum. Mol. Genet..

[16] Love M.I., Huber W., Anders S. (2014). Moderated estimation of fold change and dispersion for RNA-seq data with DESeq2. Genome Biol..

[17] Benjamini Y., Hochberg Y. (1995). Controlling the False Discovery Rate: A Practical and Powerful Approach to Multiple Testing. J. R. Stat. Soc. Ser. B Methodol..

[18] Wu T., Hu E., Xu S., Chen M., Guo P., Dai Z., Feng T., Zhou L., Tang W., Zhan L. (2021). clusterProfiler 4.0: A universal enrichment tool for interpreting omics data. Innovation.

[19] Yu G., Wang L.-G., Han Y., He Q.-Y. (2012). clusterProfiler: An R package for comparing biological themes among gene clusters. OMICS J. Integr. Biol..

[20] Kolde R., Pheatmap: Pretty Heatmaps_ (2019). R Package Version 1.0.12. https://rdrr.io/cran/pheatmap/.

[21] Gu Z., Eils R., Schlesner M. (2016). Complex heatmaps reveal patterns and correlations in multidimensional genomic data. Bioinformatics.

[22] Yu G. (2023). Enrichplot: Visualization of Functional Enrichment Result. https://www.bioconductor.org/packages/devel/bioc/manuals/enrichplot/man/enrichplot.pdf.

[23] Patel A., Yamashita N., Ascaño M., Bodmer D., Boehm E., Bodkin-Clarke C., Ryu Y.K., Kuruvilla R. (2015). RCAN1 links impaired neurotrophin trafficking to aberrant development of the sympathetic nervous system in Down syndrome. Nat. Commun..

[24] Pinto B., Morelli G., Rastogi M., Savardi A., Fumagalli A., Petretto A., Bartolucci M., Varea E., Catelani T., Contestabile A. (2020). Rescuing Over-activated Microglia Restores Cognitive Performance in Juvenile Animals of the Dp(16) Mouse Model of Down Syndrome. Neuron.

[25] Raveau M., Polygalov D., Boehringer R., Amano K., Yamakawa K., McHugh T.J. (2018). Alterations of in vivo CA1 network activity in Dp(16)1Yey Down syndrome model mice. eLife.

[26] Tuttle K.D., Waugh K.A., Araya P., Minter R., Orlicky D.J., Ludwig M., Andrysik Z., Burchill M.A., Tamburini B.A., Sempeck C. (2020). JAK1 Inhibition Blocks Lethal Immune Hypersensitivity in a Mouse Model of Down Syndrome. Cell Rep..

[27] Waugh K.A., Minter R., Baxter J., Chi C., Galbraith M.D., Tuttle K.D., Eduthan N.P., Kinning K.T., Andrysik Z., Araya P. (2023). Triplication of the interferon receptor locus contributes to hallmarks of Down syndrome in a mouse model. Nat. Genet..

[28] McCray P.B., Pewe L., Wohlford-Lenane C., Hickey M., Manzel L., Shi L., Netland J., Jia H.P., Halabi C., Sigmund C.D. (2007). Lethal Infection of K18-hACE2 mice infected with severe acute respiratory syndrome coronavirus. J. Virol..

[29] Winkler E.S., Bailey A.L., Kafai N.M., Nair S., McCune B.T., Yu J., Fox J.M., Chen R.E., Earnest J.T., Keeler S.P. (2020). SARS-CoV-2 infection in the lungs of human hACE2 transgenic mice causes severe inflammation, immune cell infiltration, and compromised respiratory function. bioRxiv.

[30] Menachery V.D., Gralinski L.E., Baric R.S., Ferris M.T. (2015). New Metrics for Evaluating Viral Respiratory Pathogenesis. PLoS ONE.

[31] Blanco-Melo D., Nilsson-Payant B.E., Liu W.-C., Uhl S., Hoagland D., Møller R., Jordan T.X., Oishi K., Panis M., Sachs D. (2020). Imbalanced Host Response to SARS-CoV-2 Drives Development of COVID-19. Cell.

[32] Zhang Q., Bastard P., Karbuz A., Gervais A., Tayoun A.A., Aiuti A., Belot A., Bolze A., Gaudet A., Bondarenko A. (2022). Human genetic and immunological determinants of critical COVID-19 pneumonia. Nature.

[33] Kusters M., Manders N., de Jong B., van Hout R., Rijkers G., de Vries E. (2013). Functionality of the pneumococcal antibody response in Down syndrome subjects. Vaccine.

[34] Colvin K.L., Yeager M.E. (2017). What people with Down Syndrome can teach us about cardiopulmonary disease. Eur. Respir. Rev..

[35] Hilton J., Fitzgerald D., Cooper D. (1999). Respiratory morbidity of hospitalized children with Trisomy 21. J. Paediatr. Child Health.

[36] Davidson M.A. (2008). Primary care for children and adolescents with Down syndrome. Pediatr. Clin. N. Am..

[37] Bruijn M., van der Aa L.B., van Rijn R.R., Bos A.P., van Woensel J.B.M. (2007). High incidence of acute lung injury in children with Down syndrome. Intensiv. Care Med..

[38] Paoloni-Giacobino A., Chen H., Peitsch M.C., Rossier C., Antonarakis S.E. (1997). Cloning of the TMPRSS2 gene, which encodes a novel serine protease with transmembrane, LDLRA, and SRCR domains and maps to 21q22.3. Genomics.

[39] Botte A., Potier M.C. (2020). Focusing on cellular biomarkers: The endo-lysosomal pathway in Down syndrome. Prog. Brain Res..

[40] Fitzpatrick V., Rivelli A., Chaudhari S., Chicoine L., Jia G., Rzhetsky A., Chicoine B. (2022). Prevalence of Infectious Diseases Among 6078 Individuals With Down Syndrome in the United States. J. Patient-Centered Res. Rev..

[41] Malle L., Martin-Fernandez M., Buta S., Richardson A., Bush D., Bogunovic D. (2022). Excessive negative regulation of type I interferon disrupts viral control in individuals with Down syndrome. Immunity.

[42] Winkler E.S., Bailey A.L., Kafai N.M., Nair S., McCune B.T., Yu J., Fox J.M., Chen R.E., Earnest J.T., Keeler S.P. (2020). SARS-CoV-2 infection of human ACE2-transgenic mice causes severe lung inflammation and impaired function. Nat. Immunol..

[43] Dyken M.E., Lin-Dyken D.C., Poulton S., Zimmerman M.B., Sedars E. (2003). Prospective polysomnographic analysis of obstructive sleep apnea in down syndrome. Arch. Pediatr. Adolesc. Med..

[44] Stebbens V.A., Dennis J., Samuels M.P., Croft C.B., Southall D.P. (1991). Sleep related upper airway obstruction in a cohort with Down’s syndrome. Arch. Dis. Child..

[45] Gatzoulis M.A., Beghetti M., Landzberg M.J., Galiè N. (2014). Pulmonary arterial hypertension associated with congenital heart disease: Recent advances and future directions. Int. J. Cardiol..

[46] Saji T. (2014). Clinical characteristics of pulmonary arterial hypertension associated with Down syndrome. Pediatr. Int..

[47] Chen X.-Q., Xing Z., Chen Q.-D., Salvi R.J., Zhang X., Tycko B., Mobley W.C., Yu Y.E. (2021). Mechanistic Analysis of Age-Related Clinical Manifestations in Down Syndrome. Front. Aging Neurosci..

[48] Rodero M.P., Crow Y.J. (2016). Type I interferon-mediated monogenic autoinflammation: The type I interferonopathies, a conceptual overview. J. Exp. Med..

[49] Malle L., Bogunovic D. (2021). Down syndrome and type I interferon: Not so simple. Curr. Opin. Immunol..

[50] Tilley A.E., Walters M.S., Shaykhiev R., Crystal R.G. (2015). Cilia dysfunction in lung disease. Annu. Rev. Physiol..

[51] Piatti G., Allegra L., Ambrosetti U., De Santi M.M. (2001). Nasal ciliary function and ultrastructure in Down syndrome. Laryngoscope.

[52] Balder R., Krunkosky T.M., Nguyen C.Q., Feezel L., Lafontaine E.R. (2009). Hag mediates adherence of *Moraxella catarrhalis* to ciliated human airway cells. Infect. Immun..

[53] Look D.C., Walter M.J., Williamson M.R., Pang L., You Y., Sreshta J.N., Johnson J.E., Zander D.S., Brody S.L. (2001). Effects of paramyxoviral infection on airway epithelial cell foxj1 expression, ciliogenesis, and mucociliary function. Am. J. Pathol..

[54] Amitani R., Wilson R., Rutman A., Read R., Ward C., Burnett D., Stockley R.A., Cole P.J. (1991). Effects of human neutrophil elastase and *Pseudomonas aeruginosa* proteinases on human respiratory epithelium. Am. J. Respir. Cell Mol. Biol..

[55] Kantar A., Oggiano N., Giorgi P.L., Braga P.C., Fiorini R. (1994). Polymorphonuclear leukocyte-generated oxygen metabolites decrease beat frequency of human respiratory cilia. Lung.

[56] Wilson D.F. (2017). Oxidative phosphorylation: Regulation and role in cellular and tissue metabolism. J. Physiol..

[57] Dinnon K.H., Leist S.R., Schäfer A., Edwards C.E., Martinez D.R., Montgomery S.A., West A., Yount B.L., Hou Y.J., Adams L.E. (2020). A mouse-adapted model of SARS-CoV-2 to test COVID-19 countermeasures. Nature.

[58] Herault Y., Delabar J.M., Fisher E.M.C., Tybulewicz V.L.J., Yu E., Brault V. (2017). Rodent models in Down syndrome research: Impact and future opportunities. Dis. Models Mech..

[59] Xing Z., Li Y., Pao A., Bennett A.S., Tycko B., Mobley W.C., Yu Y.E. (2016). Mouse-based genetic modeling and analysis of Down syndrome. Br. Med. Bull..

