# Supplementary material for: SARS-CoV-2 Infection Causes Heightened Disease Severity and Mortality in a Mouse Model of Down Syndrome
Source: Biomedicines. 2024 Feb 28;12(3):543. doi: 10.3390/biomedicines12030543 (PMC10967796; doi:10.3390/biomedicines12030543)
Supplement: Supplementary file 1 [file biomedicines-12-00543-s001.zip › biomedicines-2898377-supplementary.pdf]

Supplementary Materials

SARS-CoV-2 infection causes heightened disease severity and mortality in a mouse model of Down syndrome

Roger D. Pechous[1], Priyangi A. Malaviarachchi[1], Zhuo Xing[2], Avrium Douglas[2], Samantha D. Crane[1]†, Hayley M. Theriot[1], Zijing Zhang[3], Alireza Ghaffrieh[4], Lu Huang[1], Y. Eugene Yu[2]*, Xuming Zhang[1]*

This file includes:

Supplementary Figures S1-S2

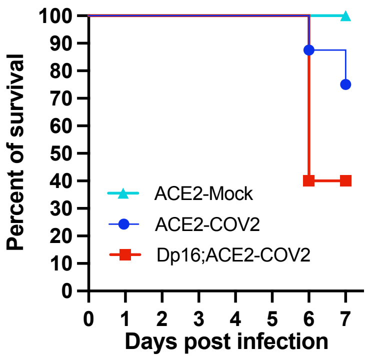

Fig. S1. Percent of survival of ACE2 and Dp16;ACE2 mice following SARS-CoV-2 infection. ACE2 and Dp16;ACE2 mice were infected intranasally with SARS-CoV-2 (COV2) at 2.5 × 10^4 PFU per mouse or mock-infected (mock) as control. The experiment was terminated on day 7 p.i. n = 4, 8, and 5 for ACE2-Mock, ACE2-COV2, and Dp16;ACE2, respectively.

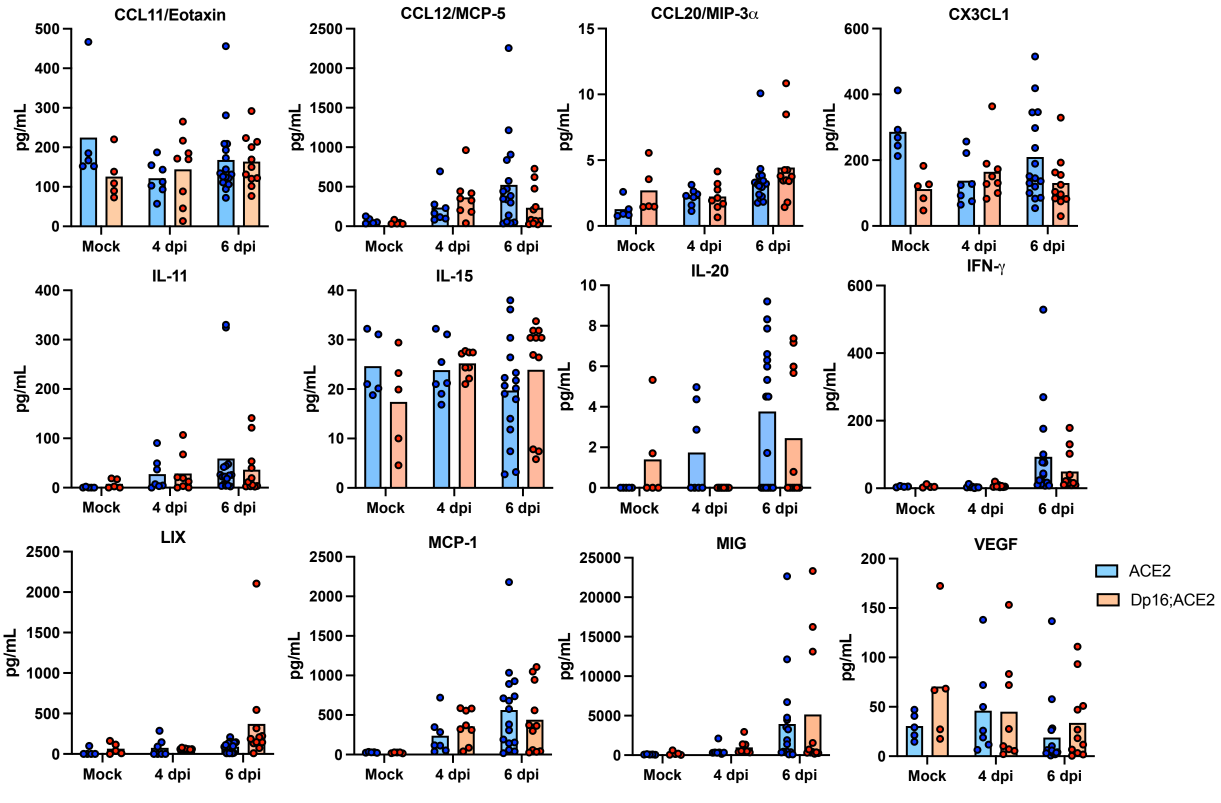

Fig. S2. Additional chemokine and cytokine responses to SARS-CoV-2 infection in the lungs of ACE2 and Dp16;ACE2 mice. Flow cytometric beads array analysis was carried out to determine the amount of cytokine and chemokine proteins in lung homogenates from ACE2 and Dp16;ACE2 mice following mock infection (Mock) or intranasal infection with SARS-CoV-2 at 2.5 × 10^4 PFU at 4 dpi and 6 dpi (4 independent experiments; n = 5, 7, and 16 for mock, 4 dpi and 6 dpi, respectively, for ACE2 mice; n = 5, 8, and 11 for mock, 4 dpi and 6 dpi, respectively, for Dp16;ACE2 mice).
